# Offering in-home video visits: a descriptive national survey of interprofessional clinicians across Veterans Health Administration

**DOI:** 10.3389/fdgth.2026.1912233

**Published:** 2026-09-14

**Authors:** Emily E. Metcalf, Da Jung Chang, Steven D. Shirk, Lauren R. Moo, Megan E. Gately

**Affiliations:** 1 Geriatric Research Education and Clinical Center, VA Bedford Healthcare System, Bedford, MA, United States; 2 Rockefeller Neuroscience Institute, West Virginia University, Morgantown, WV, United States; 3 Departments of Psychiatry and Behavioral Science and Population and Quantitative Sciences, University of Massachusetts Medical School, Worcester, MA, United States; 4 Department of Neurology, Harvard Medical School, Boston, MA, United States; 5 Department of Geriatrics, Boston University Chobanian & Avedisian School of Medicine, Boston, MA, United States

**Keywords:** attitude of health personnel, digital health, surveys and questionnaires, telemedicine, veterans health services

## Abstract

**Introduction:**

Utilization of telehealth has greatly expanded in recent years to ensure access to care during uncertain times such as the COVID pandemic. This included offering telehealth to patients not previously considered ideal candidates for telehealth. In-home synchronous video telehealth has unique knowledge and technical requirements and barriers when compared to other modalities like telephone or asynchronous websites and applications. The complexity of in-home video may limit its reach to certain patients, including older adults and those with complex medical needs, thereby exacerbating care disparities. While clinicians may be critical decision-makers in determining use of in-home video, little is known about how clinicians decide to whom video should be offered. To ensure equitable distribution of in-home video to patients who need it most, this study investigates clinician decision-making around the initial offer of in-home video and what factors influence their decision.

**Methods:**

A web-based national survey of Department of Veterans Health Affairs (VHA) interprofessional clinicians about decision-making to offer in-home video was conducted between March and April 2025. The analytic sample includes clinicians who used in-home video during the previous 12 months. Items were iteratively developed with input from interprofessional clinician subject matter experts, and gathered, 1) respondent characteristics, 2) clinical experience and video utilization details, 3) patient, clinical, and administrative factors, benefits, and barriers, and 4) video facilitators.

**Results:**

Of 11,416 respondents (5.5% response rate), most were female (68.31%), and the top three clinical disciplines included Social Workers (21.84%), Physicians (18.5%), and Nursing Professionals (15.22%). The top three clinical services delivered using in-home video were routine assessments or examinations (49.18%), assessments or examinations of new or acute issues (41.62%), and ongoing chronic disease management (40.92%). When clinicians were asked to rate the quality of care delivered via in-home video compared to in the clinic, the majority reported that they feel the quality is about the same or better when using video (84.20%). The top three patient factors that clinicians consider when deciding to offer in-home video included patient willingness and/or preference for video (77.58%), travel barriers including inclement weather, lack of transportation, traffic (76.35%), and rurality or distance from a VHA facility (74.43%). Patient factors selected the least included motor and sensory function (2,504, 21.93%), medical acuity (2,776, 24.32%), and age (3,107, 27.22%). The top three clinical and administrative factors identified as making the clinician more likely to offer in-home video included the convenience of using video to the clinician's workflow (55.23%), high patient acuity or the need to be seen quickly (55.02%), and the need for frequent follow-ups (49.21%). When clinicians were asked about the benefits of using in-home video with patients, they reported that video eliminates the need for patients to travel to and from appointments (72.80%), increased convenience for patients (70.94%), and reduced stress on patients or caregivers (60.03%). The top three most effective facilitators were automatically generated reminder emails (84.87%), reminder emails sent by the clinician or clinical staff (77.21%), and VHA-provided tablets (75.51%).

**Conclusion:**

Our findings highlight that patient-centered factors and benefits were most frequently reported by VHA clinicians as considerations when deciding to offer in-home video. Although clinician and administrative barriers were infrequently endorsed, patient-level challenges such as limited broadband access and difficulty using technology remained significant obstacles. To gain more detailed understanding, future research should explore the nuance behind clinician perspectives and potential biases in the offer of in-home video to help address the digital divide and disparities in access between subgroups of patients.

## 1 Introduction

As the largest integrated healthcare system in the United States, the Department of Veterans Health Affairs (VHA) is a unique setting for studying telehealth. VHA has been offering telehealth services for over 20 years and is the largest virtual care provider in the United States (1, 2), with over 40% of veterans receiving some form of care by telehealth (3). The Elizabeth Dole 21st Century Veterans Healthcare and Benefits Improvement Act of 2025 reaffirmed VHA's continued efforts to prioritize increased access to care through telehealth services (4), efforts which were accelerated by the COVID pandemic and resulting rapid expansion of telehealth nationwide (5–7). The public health exigency of COVID undoubtedly led to offers of telehealth to patients who may not previously have been considered ideal candidates to be seen via telehealth while simultaneously creating disparities which persist to this day. Patient, healthcare system, and clinician factors influence successful telehealth integration.

In a review of Census Bureau survey data from 2021 to 2022, trends demonstrated continued telehealth use after the COVID pandemic at higher rates compared to before the COVID pandemic started (8). However, a Digital Divide has led to inequity in use of telehealth with specific patient populations including older adults and those with complex medical needs (9, 10), creating new access barriers to healthcare. In a recent quality improvement project, VHA found that 42.7% of screened patients reported one or more unmet digital needs, which could include not having access to 1) a smartphone or computer, 2) reliable or affordable internet access or data, or 3) assistance with telehealth setup (11). Older patients, patients with dementia or higher medical complexity, and racial and ethnic minorities were more likely to report unmet digital needs. Internet access (a prerequisite for many forms of telehealth) is increasingly recognized as a “super determinant of health” (12) due to the new link between telehealth access and overall health and wellness. Social factors such as rurality, racial and ethnic minority status, unstable housing, and lower socioeconomic status exacerbate access barriers (5, 13, 14). In addition to patient-specific challenges (such as technology availability, literacy, broadband, availability of someone to help), there are potential clinician or healthcare system barriers.

At the healthcare system level, there is substantial variation in how telehealth has been integrated into clinical care based on not just clinic conditions and patient populations but also telehealth modality (6) based in part on the level of technological complexity. The terms “telehealth” and “virtual care” are often used interchangeably to describe a wide range of modalities, including asynchronous electronic consults, remote patient monitoring, and mobile or web-based applications, or synchronous telephone or video telehealth, but these modalities vary in complexity and demands. Video telehealth refers to live video visits delivered between clinic locations or into patients' homes or other choice of location. In-home video requires patients and clinicians to possess a microphone- and video-enabled device and have stable internet sufficient for video. In addition, in-home video requires the technological literacy to navigate log-in, email links, and troubleshoot potential problems, such as lost audio. The technical requirements and complexity of in-home video can lead to both patient and clinician hesitancy to offer and use video compared to other telehealth modalities such as phone. However, in-home video poses significant clinical and pragmatic benefits compared to phone, with video demonstrating improved patient-provider communication and diagnostic accuracy (15, 16). While in-home video offers distinct advantages, little is known about clinician decision-making related to offering specific forms of telehealth like in-home video with patients.

While evidence is lacking about clinician decision-making about patient appropriateness for telehealth services in general, clinicians frequently cite older age and lack of technical skills as patient barriers (17, 18). A large, nationwide, non-VHA survey of clinicians revealed that most clinicians believed telehealth is “dangerous” or an unrealistic option for older adults due to their complex medical care needs and significant physical or cognitive challenges (19). In VHA, clinicians view a patient's lack of technical skills as a barrier to providing care via telehealth (20). However, it remains unclear how clinicians are determining patient skill level or readiness for telehealth services, particularly services like in-home video which are more technologically complex. Given this knowledge gap, gathering data about the factors influencing clinicians' decision-making has been highlighted as a priority area in deploying new technologies for healthcare (21). As “gatekeepers” of telehealth, particularly the offer of in-home video (22), clinicians hold attitudes and perspectives which may be critical to ensuring equitable distribution of in-home video amongst patients who need it the most. The clinician role in facilitating access to telehealth, including a clinician's knowledge, skills, abilities, comfort, confidence, and potential bias, has also been recommended as a priority research area to increase virtual care access at VHA (23). It is unclear how VHA clinicians assess suitability for in-home video appointments, and these assessments are not standardized across clinicians, clinics, or facilities. To address these knowledge gaps, we conducted a national survey of VHA clinicians to investigate their perspectives on clinical services offered via in-home video, factors they consider when offering video, benefits and barriers of video, and the frequency and effectiveness of current VHA facilitators or resources to support video use. This study aims to increase the availability of healthcare to patients facing barriers to accessing telehealth by exploring clinician decision-making around the offer and use of in-home video. As such, the present study provides a unique snapshot of VHA clinicians' perspectives on the use of in-home video appointments, with potential implications for a broader, more stable, post-COVID world that continues to embrace and expand telehealth use.

## 2 Materials and methods

### 2.1 Study design and population

We conducted a cross-sectional nationwide web-based survey of interprofessional VHA clinicians working in outpatient settings. We utilized VHA Corporate Data Warehouse (CDW) data to identify all VHA clinicians employed across VHA's 1,298 healthcare facilities (173 medical centers and 1,199 outpatient clinic sites) (24). Specifically, we pulled data on provider type and classification of individuals with an active assignment (i.e., did not have a termination date after December 2024) and a VHA-issued email address listed in the CDW. Disciplines included physicians, physician assistants, nurse practitioners, nursing professionals, psychologists, pharmacists, nutritionists/dieticians, dentists, social workers, and rehabilitation professionals (occupational therapy, physical therapy, kinesiotherapy, recreation therapy, and speech language). Duplicate email addresses were removed. Criteria for participation in the survey included, a) identifying as a clinician (or fellow, resident, or trainee who has trained full time at VHA for at least a month), and b) delivering care in an outpatient setting. No other eligibility criteria were applied. The current paper reports results from a subset of the total respondents who reported delivering care via video in the 12 months prior to survey launch. Respondents who reported not using video were asked a different series of questions that are not reported here. The survey was conducted between March 4 and April 9, 2025.

### 2.2 Survey development and measures

Survey items gathered data on clinician demographics (including practice settings and services delivered using in-home video), perceived clinical, patient, and administrative factors influencing the decision to offer in-home video, perceived quality of video visits, and perceived benefits and barriers to in-home video utilization. Survey items were iteratively developed by the study team and were informed by interviews with VHA Subject Matter Experts (SMEs) across a variety of the disciplines which were part of the target recruitment pool (please see Gately et al. 2026 for complete details of interviews and interview findings which informed survey development) (25). The survey draft was sent to SMEs for either, a) asynchronous feedback via email, or b) synchronous feedback via video conferencing on the scope, clarity, and length of the survey, with the built survey reflecting their feedback. Once the survey was built in REDCap (Research Electronic Data Capture), an electronic data capture tool hosted at VHA, a subset of SMEs who completed interviews (*n* = 8) and 8 internal VHA team members (clinicians and non-clinician staff with experience developing and launching REDCap surveys for clinicians) further piloted the web-based survey for quality assurance of survey links, branching logic, and other REDCap functionality. The average survey completion time was 15 min.

#### 2.2.1 Survey items

The final survey included 32 items with the 18 items administered to the subgroup of respondents who had delivered care via in-home video in the 12 months prior to the survey described below. See Supplementary File 1 for the survey. Questions were a mix of Likert scale, select-all-that-apply, and open text options. Five items addressed inclusion criteria, including consent to participate, clinical discipline (confirming that the respondent identifies as a clinician, and if they are a fellow, resident, or trainee, that they have been training at VHA full-time for at least one month), if they deliver care in an outpatient setting (including what percentage of care they deliver in an outpatient setting). Three items addressed in-home video utilization, including percentage of care delivered using video, types of video clinical services, and perceived equivalence in quality of care delivered via video compared to in-clinic. One item asked clinicians about patient factors that were considered when offering in-home video, with respondents given a list where they could select all that apply. One item asked about clinical or administrative factors influencing the decision to offer in-home video, and respondents rated the level of influence of each factor (positively-neutral-negatively). Some of the options listed here are specifically available at VHA, including having a Telehealth Clinical Technician (TCT) available to help with scheduling, test calls, or technical assistance.

To evaluate barriers to in-home video, one item presented a list of several administrative (four options), patient (five options), and clinician (four options) barriers of which respondents were asked to select all barriers that they have encountered (they could select all that apply). One item asked clinicians about the perceived benefits of in-home video. Two items were used to assess the frequency of use and the effectiveness of current VHA resources that support in-home video. Many of these resources are unique to VHA, including a national VHA virtual care Help Desk, Digital Divide Consults for patients without reliable internet or an internet-connected device, VHA-loaned devices (internet-connected tablets provided at no cost to the veteran), a national VHA telehealth website, and VHA-developed telehealth handouts and instruction guides (2). Respondents also completed four demographics questions, including years of practice as a clinician in VHA and their age and sex. For most items, options to select Unsure or Other were provided, with corresponding optional open text boxes.

### 2.3 Recruitment and data collection

Survey invitations were sent to 286,734 VHA clinicians, involving an initial email followed by three follow-up reminder emails over the survey period. Respondents accessed the survey through a secure link emailed directly to them that was only accessible while logged into an active VHA network account. Respondents were able to review their answers using the “back” button. Data were collected and managed using REDCap, a secure, web-based application designed to support data capture for research studies, providing 1) an intuitive interface for validated data entry; 2) audit trails for tracking data manipulation and export procedures; 3) automated export procedures for seamless data downloads to common statistical packages; and 4) procedures for importing data from external sources. Due to policy restrictions, participants were not compensated for participation.

### 2.4 Data analysis

Survey data were exported from REDCap into both Microsoft Excel and SAS 9.4 and summarized using frequencies and percentages. All available data were used for each variable reported. For each question, percentages were calculated using only respondents who provided an answer. All surveys with completed eligibility questions were included in analysis; however, since item completion was not required, response numbers vary and are reported by question. Some Likert scales were collapsed (e.g., combining Often-Always and Rarely-Never) for ease of presenting results.

### 2.5 Ethics statement

This project was reviewed by the Institutional Review Board of the VHA Bedford Healthcare System and determined to be non-research, as this project evaluated ongoing clinical programming for quality improvement. Though the survey was non-research, all study procedures were in accordance with the ethical standards of relevant institutional or national bodies and consistent with the revised Helsinki Declarations. Specifically, surveys included statements related to the purpose of the study, the voluntary nature of participation, respondents' right to stop the survey at any time, and the handling of their data in accordance with VHA regulatory procedures. Respondents were provided contact information for the study team before beginning the survey to address questions, concerns, and feedback. This study was also reviewed and approved by VHA's Organizational Assessment Sub-Committee (OASC) and Office of Labor - Management Relations (LMR), as part of standard procedures for employee surveys.

## 3 Results

### 3.1 Respondent characteristics

Of the 286,734 VHA clinicians invited to participate, 20,325 clicked on the survey link, 19,913 consented to participate, and 15,433 (5.4% response rate) met eligibility requirements. Of those eligible respondents, 11,416 (74.0%) reported using in-home video in the last 12 months and were included in analysis (see Figure 1 for survey participant flow diagram). Table 1 displays respondent demographics. Most respondents were female (5,724/8,380, 68.31%). Regarding age, of 8,466 responses, 2,619 (30.94%) reported being 45–54 years, 2,312 (27.31%) reported being 35–44 years, and 2,088 (24.66%) reported being 55–64 years. Among the 11,416 respondents, the top three clinical disciplines included Social Workers (2,493, 21.84%), Physicians (2,112, 18.50%), and Nursing Professionals (RN, LPN, etc.; 1,738, 15.22%). Among the 2,112 physicians, the top three specialties identified included Psychiatry (433, 21.53%), Primary Care (320, 15.91%), and Internal Medicine (279, 13.87%). Supplementary File 2 includes the complete list and distribution of respondents' clinical disciplines and physician specialties.

**Figure 1:**
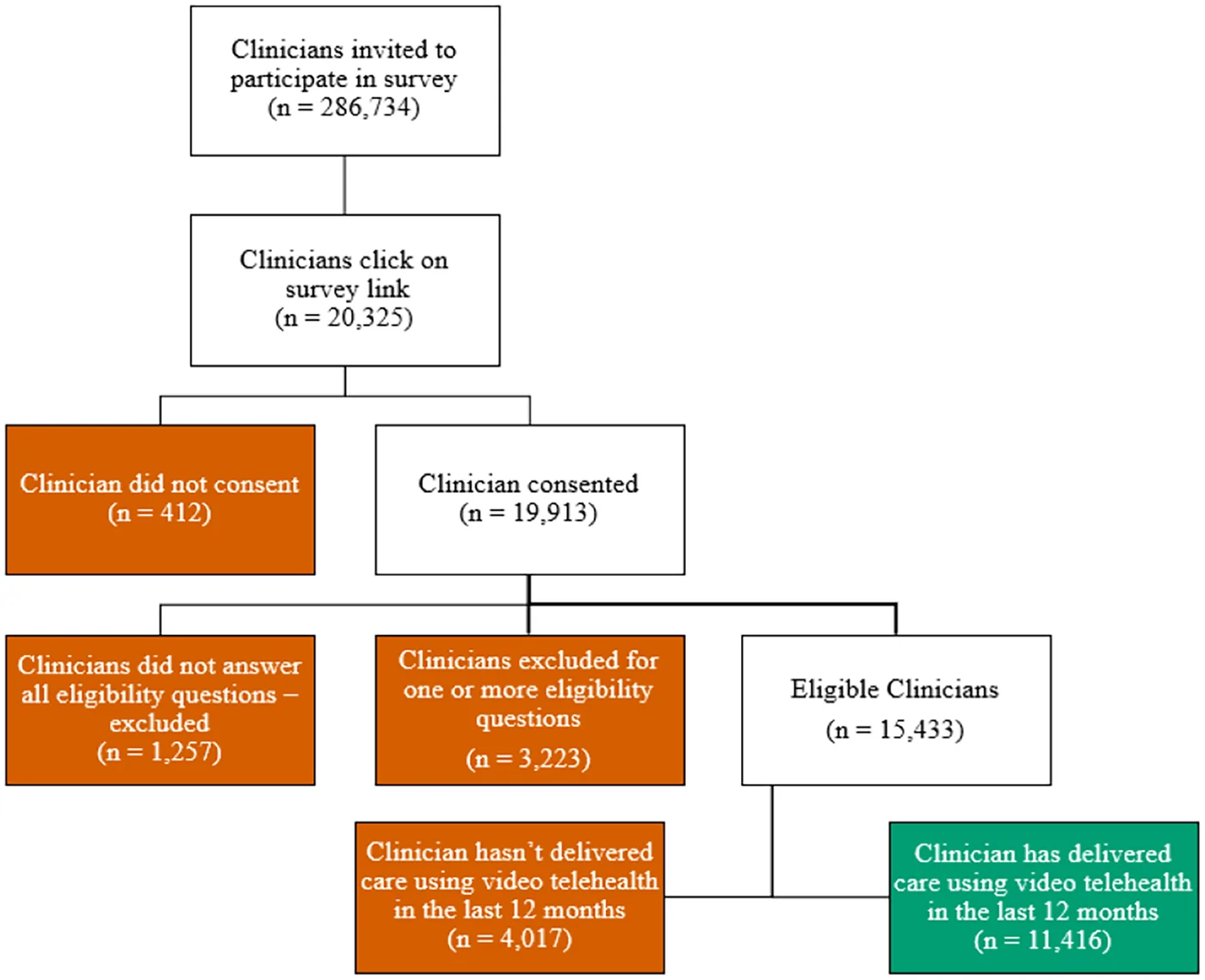
Survey participant flow diagram.

**Table 1:** Respondents' demographics.

Demographic Variables	N	Responses, *n* (%)
Age	8,466	
18–24		11 (0.13)
25–34		862 (10.18)
35–44		2,312 (27.31)
45–54		2,619 (30.94)
55–64		2,088 (24.66)
65–74		515 (6.08)
75–84		55 (0.65)
85+		4 (0.05)
Sex	8,380	
Female		5,724 (68.31)
Male		1,783 (21.28)
Prefer not to answer		873 (10.42)
Years as a Clinician	8,619	
< 1		74 (0.86)
1–5		1,106 (12.83)
6–10		1,444 (16.75)
11–20		2,853 (33.10)
21–30		2,028 (23.53)
> 30		1,114 (12.92)
Years as a Clinician at VHA	8,612	
< 1		400 (4.64)
1–5		3,017 (35.03)
6–10		2,128 (24.71)
11–20		2,367 (27.48)
21–30		533 (6.19)
> 30		167 (1.94)

Not all questions were required, creating variation in the sample size for each question.

### 3.2 Clinical experience and video utilization

Most respondents had more than 10 years of experience as a clinician (5,995/8,619, 69.56%) but had 10 years or less experience as a clinician working at VHA (5,545/8,612, 64.39%). The majority of respondents (7,771/11,416, 68.07%) only deliver care in an outpatient setting, with another 2,277 (19.95%) respondents delivering care in an outpatient setting over half of the time. According to 11,416 respondents, there was a broad range in the percentage of care delivered via in-home video over the last 12 months, with 3,552 (31.11%) respondents using video 1%–9% of the time, 2,744 (24.04%) using video 10%–24% of the time, 2,231 (19.54%) using video 25%–50% of the time, 2,278 (19.95%) using video 51%–99% of the time, and 611 (5.35%) using video 100% of the time. The top three clinical services delivered using in-home video were routine assessments or examinations (5,614, 49.18%), assessments or examinations of new or acute issues (4,751, 41.62%), and for ongoing chronic disease management (4,671, 40.92%). Related to other specific types of care being delivered, in-home video was often used for care coordination (3,516, 30.80%), medication management (3,364, 29.47%), and home environment assessment or observation (1,292, 11.32%). See Table 2 for the frequency of clinical services delivered using in-home video. Clinicians were asked to rate the perceived quality of care delivered via in-home video compared to in the clinic, with the majority reporting that the quality is about the same or better when using video (7,522/8,933, 84.20%, see Figure 2).

**Table 2:** Clinical services delivered using video telehealth.

Clinical Services	Responses, *n* (%)
Routine assessment/examination	5,614 (49.18)
Assessment/examination of new or acute issue	4,751 (41.62)
Ongoing chronic disease management	4,671 (40.92)
Ongoing care of mental health issues (e.g., counseling or talk therapy)	4,318 (37.82)
One-to-one care	3,930 (34.43)
Care coordination	3,516 (30.80)
Medication management	3,364 (29.47)
Group treatment	2,334 (20.44)
Hospital or ED follow-up care	1,909 (16.72)
Home environment assessment or observation	1,292 (11.32)
Other	701 (6.14)
None	258 (2.26)

Respondents could select more than one answer, so totals do not add up to 100%.

**Figure 2:**
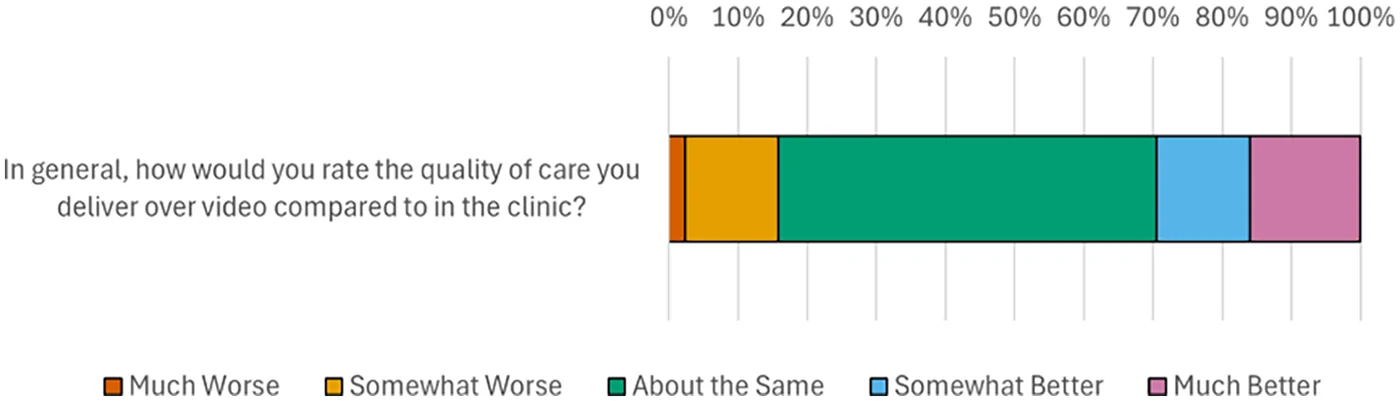
Quality of video telehealth.

### 3.3 Patient, clinical, and administrative factors, benefits, and barriers

Clinicians were asked to identify the patient factors that they consider when deciding whether to offer an in-home video appointment. The top three factors (reported by our 11,416 respondents) included patient willingness and/or preference for video (8,857, 77.58%), travel barriers including inclement weather, lack of transportation, traffic (8,716, 76.35%), and rurality or distance from a VHA facility (8,497, 74.43%). Patient factors that were selected the least when considering offering in-home video included patient motor and sensory function (e.g., strength, range of motion or ROM, coordination, sensation; 2,504, 21.93%), patient medical acuity (2,776, 24.32%), and patient age (3,107, 27.22%). See Table 3 for all patient factors identified by respondents.

**Table 3:** Patient factors.

Patient Factors	Responses, *n* (%)
Willingness and/or preference for video	8,857 (77.58)
Travel barriers including inclement weather, lack of transportation, traffic	8,716 (76.35)
Rurality or distance from a VHA facility	8,497 (74.43)
Skills with technology or technological literacy	7,759 (67.97)
Access to email, device (e.g., laptop or smartphone), or broadband internet	7,693 (67.39)
Patient/caregiver history of successful video encounters	6,628 (58.06)
Cognition	6,083 (53.28)
Vision and hearing	5,661 (49.59)
Ability to communicate effectively	5,390 (47.21)
Availability of a caregiver to provide assistance	4,927 (43.16)
Functional status (ADL and IADL status)	4,771 (41.79)
Psychological/psychiatric factors (e.g., mood, PTSD, substance use disorder)	3,887 (34.05)
Medical complexity	3,492 (30.59)
Age	3,107 (27.22)
Medical acuity	2,776 (24.32)
Motor and sensory function (e.g., strength, ROM, coordination, sensation)	2,504 (21.93)
Other	360 (3.15)
None	142 (1.24)

Respondents could select more than one answer, so totals do not add up to 100%.

Clinicians were also asked to identify the clinical and administrative factors that influence their decision to offer in-home video. The top three clinical and administrative factors identified as making the clinician more likely to offer in-home video included the convenience of using video to the clinician's workflow (4,871/8,820, 55.23%), high patient acuity or the need to be seen quickly (4,862/8,836, 55.02%), and the need for frequent follow-ups (4,353/8,846, 49.21%). None of the factors were strongly identified as a negative factor, i.e., one that made the clinician less likely to offer video. Of note, the top negative factor, high patient acuity or the need to be seen quickly (932/8,836, 10.55%), was also identified as a top positive factor. Interestingly, there were four factors that more than half of respondents selected as neutral or not a factor in offering in-home video. These included: 1) number of patients in the clinician's panel (6,063/8,802, 68.88%), 2) departmental or facility targets for number of video encounters (5,470/8,707, 62.82%), 3) workload credits (5,193/8,769, 59.22%), and 4) number of available video appointments (5,054/8,781, 57.56%). Of note, staff availability to provide technical support was the most frequent factor reported as not available to clinicians (1,989/8,816, 22.56%). Figure 3 displays the distribution of respondents' evaluations of each clinical and administrative factor.

**Figure 3:**
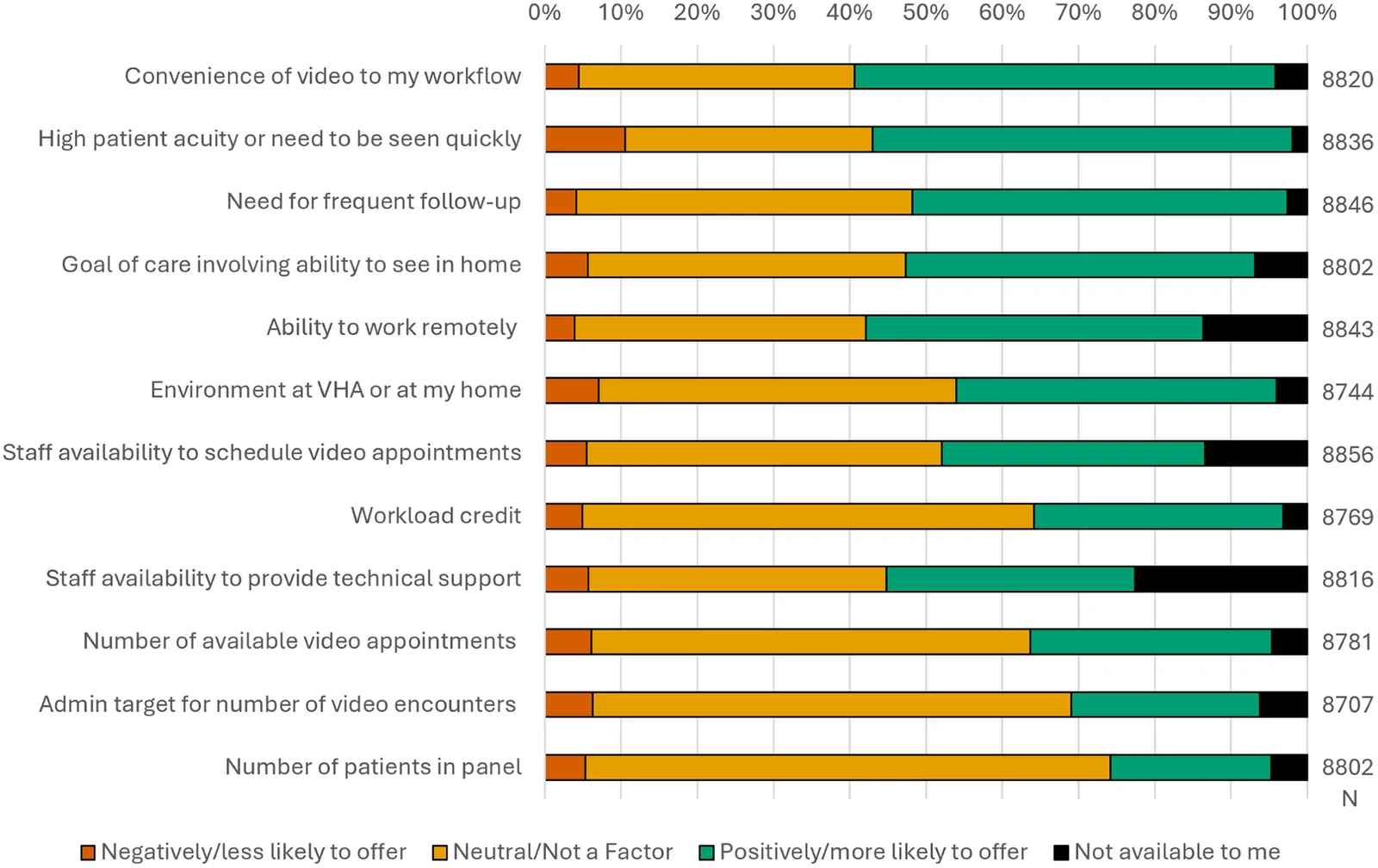
Clinical and administrative factors.

Clinicians were asked if they had encountered specific clinician, administrative, or patient barriers to integrating in-home video into their practice. The most common response for both clinician and administrative barriers (of 11,416 respondents) was none (7,634, 66.87%, and 5,111, 44.77%, respectively). The next most common clinician barriers of those listed were that the clinical care being provided is not feasible over video (992, 8.69%), and the clinician saw little added benefit to using video (771, 6.75%). The next most common administrative barriers were technical challenges on the clinician or VHA side (3,062, 26.82%) and inadequate space, physical locations, and/or related equipment (1,749, 15.31%). The top three patient factors that were identified as barriers to in-home video included patient or caregiver difficulty with technology limiting their capacity to use video (7,684, 67.31%), patient lack of access to broadband/Wi-Fi, email, or the device needed for video (4,956, 43.41%), and patient preference for telephone instead of video (4,936, 43.24%). See Table 4 for the full list of barriers to in-home video use. When clinicians were asked about the benefits of using in-home video with patients, they reported that video eliminates the need for patients to travel to and from appointments (8,311, 72.80%), increased convenience for patients, e.g., reduces need to take time off work (8,099, 70.94%), and reduced the stress on patients or caregivers (6,853, 60.03%). See Table 5 for all evaluated benefits of in-home video use.

**Table 4:** Barriers to video telehealth use.

Barriers	Responses, *n* (%)
Administrative Factors	
None	5,111 (44.77)
Technical challenges on the clinician/VHA side	3,062 (26.82)
Inadequate space, physical locations, and/or related equipment	1,749 (15.31)
Lack of administrative support (e.g., assistance with scheduling, setting up clinics, etc.)	1,675 (14.67)
Other	1,030 (9.02)
Lack of leadership support at supervisor or facility level for using video	697 (6.11)
Patient Factors	
Patient/caregiver difficulty with technology limiting their capacity to use video	7,684 (67.31)
Patient lack of access to broadband/Wi-Fi, email, or device needed for video	4,956 (43.41)
Patient preference for telephone instead of video	4,936 (43.24)
Patient/caregiver preference for coming into the clinic	3,898 (34.15)
Patient/caregiver medical/functional impairments limiting their capacity to use video	3,226 (28.26)
None	1,431 (12.54)
Other	431 (3.78)
Clinical Factors	
None	7,634 (66.87)
The clinical care I provide is not feasible over video	992 (8.69)
Other	886 (7.76)
I see little added benefit to using video	771 (6.75)
I found the training or other set-up requirements burdensome	663 (5.81)
I lack the time and/or capacity to adapt my clinical procedures to video	321 (2.81)

Respondents could select more than one answer, so totals do not add up to 100%.

**Table 5:** Benefits to video telehealth use.

Benefits	Responses, *n* (%)
Eliminates the need for patients to travel to and from appointments	8,311 (72.80)
Increased convenience for patients (e.g., reduces need to take time off work)	8,099 (70.94)
Reduces the stress on patients or caregivers	6,853 (60.03)
Enables patients faster access to care	6,630 (58.08)
Reduces potentially harmful exposures (viruses, infectious diseases)	6,055 (53.04)
Enables me a view into patients' homes or of patients in-home functioning	4,284 (37.53)
Allows me to provide more comprehensive care to patients	3,955 (34.64)
Other	494 (4.33)
None	162 (1.42)

Respondents could select more than one answer, so totals do not add up to 100%.

### 3.4 Video facilitators

Clinicians were asked how often they use (or the veterans that they serve use) different supports for in-home video. The top three facilitators used by clinicians included automatically generated reminder emails (6,410/8,935, 71.74% selected often or always), reminder emails sent by the clinician or clinical staff (3,719/8,900, 41.79% selected often or always), and the clinician's assistance with technical issues during log-in to the video appointment (2,264/8,857, 25.56% selected often or always). The least used facilitator was test calls conducted by the clinician (5,650/8,899, 63.49% selected never or rarely). For each facilitator that a respondent reported that they used, they were also asked how effective that facilitator was for enabling in-home video appointments. The top three most effective facilitators (responses of effective, very effective, or extremely effective) were automatically generated reminder emails (6,008/7,079, 84.87%), reminder emails sent by the clinician or clinical staff (4,489/5,814, 77.21%), and VHA-provided tablets (4,422/5,856, 75.51%). See Figure 4 for the distributions of responses for facilitator usage (panel A) and facilitator effectiveness (panel B). Of note, clinicians who reported not using a particular facilitator were not asked about its effectiveness.

**Figure 4:**
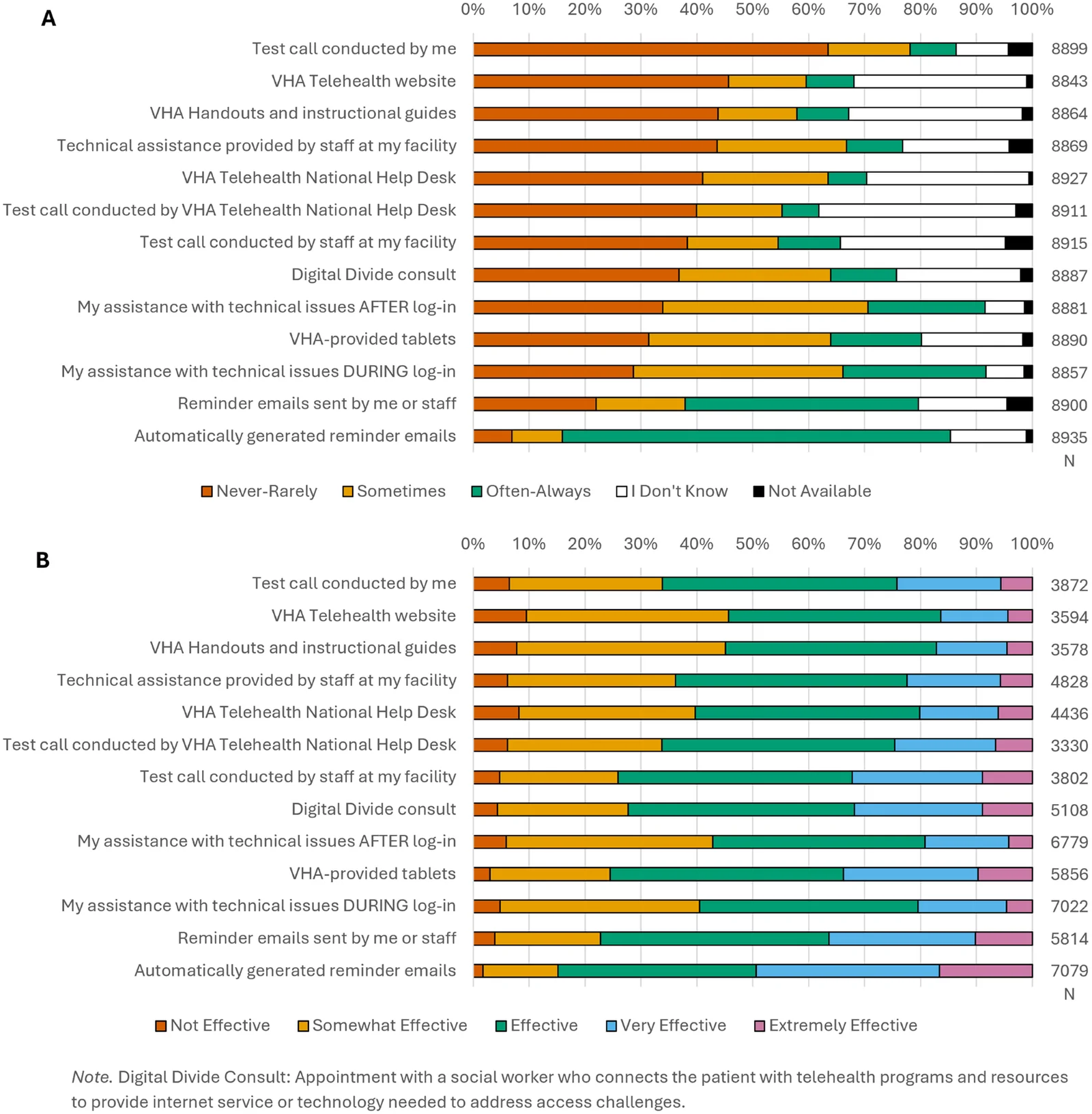
Video telehealth facilitators: panel **(A)**. Usage Frequency. Panel **(B)**. Effectiveness.

## 4 Discussion

In this nationwide survey, we explored interprofessional VHA clinicians' perspectives on in-home video, including types of services utilized and perceived quality relative to in-clinic care. We also captured multi-level factors influencing the offer of in-home video, including barriers, benefits, and the perceived usefulness of supportive VHA resources. See below for Key Findings and Contextualization of Findings by topic area.

### 4.1 Key findings

This national sample of clinicians using live in-home video from across VHA, the largest integrated healthcare system in the United States, reveals current patterns integrating video-based care into clinical practice. Clinicians across a wide range of disciplines utilize in-home video, particularly social work, behavioral health, and primary care. Despite most respondents having extensive clinical experience, many were relatively new to VHA, suggesting that video adoption is occurring across experience levels. In-home video was used for diverse clinical services, extending well beyond follow-ups to include assessments for routine and acute issues and for chronic disease management. Significantly, most clinicians perceived the quality of video-delivered care to be comparable to in-clinic care, reinforcing the growing acceptability of in-home video as a standard modality within VHA. Clinicians' decisions to offer in-home video were most frequently reported as influenced by patient-centered considerations, especially patient preference, transportation challenges, and geographic distance. Although clinician and administrative barriers were infrequently endorsed, patient-level challenges such as limited broadband access and difficulty using technology remained significant obstacles. Concurrently, clinicians identified clear benefits of in-home video care, including reduced travel burden and improved convenience. Facilitators such as automated reminders and VHA-provided tablets were consistently viewed as effective, underscoring the importance of system-level supports in sustaining in-home video use.

### 4.2 Contextualization of findings

#### 4.2.1 Patterns of in-home video utilization

This study's finding that the types of clinical services being delivered by VHA clinicians using in-home video are wide-ranging and diverse aligns with extant literature about video for neurological examination (26–28) and physical and/or motor assessment (29–31). Similarly, our finding that clinicians used in-home video for medication management (32–34) and environmental assessment also aligns with extant literature (35–37). This latter finding suggests that video care into patients' homes may provide a window into patients' day-to-day lives not presently possible when only seeing patients in the clinic or when using a telehealth modality that lacks a visual component, such as phone. In-home video may even lead to improved outcomes; for example, a study examining the impact of a telemedicine tertiary care clinic that included video services found an association between use of virtual care and medication adherence (38). Related to the use of video to address home safety, our own work delivering video-based home safety assessments to caregivers of patients with dementia (36, 37) and another recent study with a similar population (39) found video-based evaluation is feasible and acceptable with a population at high risk of falls and other negative outcomes. Similarly, a recent study has validated a structured approach for delivering video-based home safety assessments (40), expanding the evidence base for more complex video-based assessments. These findings may be particularly beneficial to older patients wishing to age-in-place.

#### 4.2.2 Influence of patient acuity on in-home video utilization

Our finding that acute issues positively influence use of in-home video raises interesting questions related to clinician decision-making. Video is often perceived as limited for addressing acute patient needs, in part due to perceived inability to conduct certain diagnostics like a physical examination, vitals, and labs (41). Video is also perceived as limited for more severe psychiatric cases, such as patients with psychosis or paranoia (42). This may relate to perceived safety risks involved with video, due to a lack of standardized safety protocols for video-based care (43). On the other hand, a recent study of family medicine visits conducted by telehealth to address acute care needs revealed the benefits of video at reducing the need for in-person visits (44). Similarly, a recent policy brief from the American Medical Association highlighted the association between video and reduced emergency department visits, demonstrating potential benefits of in-home video to care coordination and planning. Clinician and healthcare system logistics, including clinical discipline and local scheduling constraints, may also influence decision-making. For example, one clinician may have the scheduling flexibility to quickly add in an extra patient for a highly acute case and thus see video telehealth as a positive, while specialty disciplines may schedule so far out that they feel that anything acute needs to be seen in urgent care or in an emergency room. Of note, the shift to virtual care is often undertaken by clinicians in the absence of guidelines for delivering clinical care over video. Though guidelines for video-based delivery of services such as neurology (45) and physical examination (46, 47) have expanded recently, there remains a lack of evidence-based guidance for standardizing care over video. In general, clinicians' willingness to innovate in the absence of explicit and universal guidelines suggests the potential for long-term integration and expansion of video-based care to address patient access needs. More research is needed to fully understand how factors such as patient acuity influence clinician decision-making, including analysis by clinician discipline and type of care. Facilitators and barriers identified via research can be integrated into evidence-based guidelines to facilitate further expansion of video-based care.

#### 4.2.3 Perceived quality of video care

Our finding that clinicians perceive the quality of care delivered via in-home video equal to or better than in-clinic care aligns with a growing body of literature comparing care between service delivery modes. A systematic review and meta-analysis of randomized control trials of telehealth-delivered psychotherapy (most of which were video-based) found telehealth and face-to-face therapy produced comparable results across all measured outcomes, with no significant differences in symptom severity or overall improvement, nor in functional outcomes, therapeutic alliance, or patient satisfaction (48). Another study of primary care visits comparing telehealth (video or phone) to in-clinic found that telehealth resulted in slightly more in-person follow-up visits and that there was rough equivalence between telehealth-based prescribing (49). Similarly, a study of telehealth (video or phone) for patients with heart failure found no difference in the likelihood of a hospitalization after a phone or video visit compared to an in-person visit, and that video visits were associated with fewer hospitalizations overall (50). Thus, while more research is needed comparing the impact of video versus in-clinic care on both short and long-term patient outcomes, our finding that clinicians perceive video as being of comparable quality is reassuring.

Related to the perceived value of in-home video, clinicians in our survey may hold a more sanguine view than other studies reporting clinician and patient perspectives suggest. While patients and clinicians have demonstrated an openness to video, many studies indicate a preference for in-clinic due to its perceived superior quality and comprehensiveness (51, 52), or because of technological limitations or challenges (53). Preferences, however, are mixed, with one study reporting that when remote care options are available, patients may choose an earlier remote visit with a nonreferring clinician over a later in-clinic appointment with their referring clinician, particularly for acute or worsening symptoms. This finding suggests a context-specific approach to care modalities (54). When discussing availability of telehealth for behavioral health, patients often report either not being given a choice or a perception that clinicians did not value their preferences (55), suggesting that clinicians should try to elicit, and where possible meet, patient preferences for service delivery options.

#### 4.2.4 Determinants of in-home video utilization and associated benefits and barriers

The primary factors determining in-home video use reflected patients' logistical and access needs versus medical status or age. This reveals a patient-focused approach common to the VHA (56–59), reflecting VHA's longstanding commitment to integrate telehealth services into veteran care. Specific patient factors influencing clinician decision-making included patient distance from care, underscoring travel barriers faced by the sizable proportion of VHA patients residing in rural areas (60, 61). Relatedly, clinicians' perceived benefits of in-home video were similarly patient-centric, including increased convenience and reduced need for travel, both of which are also valued by patients in extant studies (62, 63). Clinicians' perception that video may reduce caregiver stress aligns with systematic reviews of telehealth for caregivers of older adults with various health conditions (64) and for caregivers of palliative care patients (65). These findings suggest that clinicians should consider the value of video on not just patients but also patients' family members, particularly adult child caregivers of older patients for whom video may be particularly convenient due to competing responsibilities from work and family (66).

The fact that patient health and age were less influential on clinicians' offer of in-home video is especially salient given the large proportion of VHA patients who are high-risk, high-need due to age and medical complexity (67). Such patients may benefit from video the most; in fact, a recent review of the impact of telehealth on health outcomes for older adults found that video interventions could help to improve physical function, manage chronic disease, and reduce avoidable hospitalizations (68). On the other hand, the fact that clinicians report patient or caregiver difficulty with technology as the primary barrier to in-home video aligns with extant data demonstrating the importance of considering caregivers' own technical support needs in offering digital health solutions such as video (69). This also aligns with data recommending caregiver involvement in the development and planning of technological solutions (70, 71). Our own prior qualitative work examining the factors influencing in-home video for dementia management underscores the centrality of caregivers (72), revealing patient cognitive status as a key factor influencing caregiver involvement in video. In addition, caregivers may be more apt to participate in certain types of clinical care; for example, our prior work and the work of others indicates that caregivers may be particularly helpful to assist in occupational therapy services (73, 74), which often rely on visualizing patients and their environments. The low endorsement of age should not be interpreted as evidence that age-related barriers or gatekeeping are absent. Technology literacy, cognition, sensory function, broadband access, and caregiver availability may indirectly reflect age, disability, or socioeconomic barriers. However, our finding that VHA clinicians consider patient preference and need more frequently than age is an encouraging sign towards potentially reducing the noted digital divide.

Our finding that patients' limited access to technological resources for in-home video as a key barrier is particularly relevant given VHA's substantial telehealth infrastructure, which includes access to VHA-issued tablets and facility technical support teams (5, 75). The fact that some clinicians stated that technical support was not available to them may suggest clinician knowledge gaps about available resources or that resources may differ across care locations*.* This also underscores that even in the best of circumstances—such as in VHA—broader infrastructure challenges like lack of broadband availability, particularly in rural areas (76), may constrain video. Similarly, the recent finding about veterans' unmet digital needs (e.g., not having a video-capable smartphone or tablet or Wi-Fi sufficient for video) (11), aligns with the finding from outside VHA that older patients, patients with dementia or higher medical complexity, and racial and ethnic minorities are more likely to report digital needs. This speaks to broader access challenges for specific populations. Related to system-level factors, our finding that automatically generated email reminders were facilitative to in-home video suggests the potential for telehealth resources to streamline workflows for clinicians and patients.

### 4.3 Strengths and limitations

The current study boasts several key strengths. First, we achieved an impressively large survey sample size of 11,416 respondents nationwide, which offers highly interprofessional perspectives regarding utilization of in-home video that are potentially generalizable across medical professions. Second, this project exclusively studies live in-home video visits versus other forms of telehealth which are less complex. We prioritized the telehealth modality that both patients and clinicians have reported challenges with and hesitancy to use, whereas much of the literature on telehealth is either broader or includes modalities like phone which are less complex, making results difficult to interpret and operationalize for system improvements. Another key strength is that this study is evaluating clinical decision-making around the offer of in-home video within VHA, an integrated healthcare system with the unique policies, supports, and structure provided to expand access to healthcare via telehealth. This could potentially limit the generalizability of our findings, given that VHA's broad telehealth support resources such as Digital Divide consults may not be present in other settings. Additionally, having access to VHA's extensive telehealth infrastructure could impact clinician attitudes towards telehealth, or may attract clinicians that view telehealth more favorably or have more experience using telehealth. However, resources that VHA providers find useful may be effective strategic targets for infrastructure development within community telehealth environments in other healthcare systems. Selection bias and non-response were limitations, given the relatively low (5.5%) response rate. This study used a convenience sample and like all studies using a convenience sample, selection bias could be an issue. Because we lack comparable data on non-respondents, the direction and magnitude of any bias cannot be determined. Additionally, we were unable to compensate participants for their time, despite evidence demonstrating that payment incentives increase web survey response rate (77).

Future directions for analysis include comparison with clinicians who were non-users of in-home video (not included in this study), and sub-group analyses of regular versus infrequent use of video, including a breakdown by discipline and care type, on clinician perspectives on offering video. These exploratory sub-group analyses, complemented by quantitative measures of how frequently different in-home video telehealth facilitators and resources are utilized and by whom, would further expound on the nuance behind clinician perspectives and potential biases in the offer of video. In addition, future work should examine how clinicians weigh competing factors during decision-making. It is critical to gain more detailed understanding regarding the circumstances in which clinicians offer video to help bridge systematic gaps in access and address disparities in access between subgroups of patients. Relatedly, gathering patient perspectives about how and when they receive an offer for video telehealth would contribute to our understanding.

## 5 Conclusion

This study offers important insights into current clinical practices and trends as well as factors influencing clinician decision-making around the offer of in-home video, highlighting potential avenues to optimize video utilization going forward. VHA clinicians utilize in-home video for a variety of clinical services, including initial assessment, follow-up, and care coordination, suggesting that in-home video can effectively support patients across a continuum of care. This study also reveals that clinicians consider patients' logistical and access needs more frequently than age or medical status when offering in-home video, potentially benefiting rural and high-risk patients. According to clinician respondents, in-home video may also offer a valuable window into patients' day-to-day lives, which can add clinical value to services delivered in patients' homes or choice of location. Barriers to broadscale video implementation, such as access to necessary devices, broadband limitations, and patient, caregiver, and even clinician technical support needs, underscore the importance of a multi-pronged implementation strategy that involves enhancing infrastructure while also developing targeted education and training for hesitant or less technologically savvy patients, caregivers, and clinicians alike.

## Data Availability

The datasets presented in this article are not readily available because due to privacy, confidentiality, and security concerns, this dataset is only available via request. To gain access, data requestors will need to sign a data access agreement. Requests to access the datasets should be directed to Megan E. Gately, megan.gately@va.gov.
