# Supplementary material for: Offering in-home video visits: a descriptive national survey of interprofessional clinicians across Veterans Health Administration
Source: Front Digit Health. 2026 Sep 14;8:1912233. doi: 10.3389/fdgth.2026.1912233 (PMC13617108; doi:10.3389/fdgth.2026.1912233)
Supplement: Supplementary file 1 [file Supplementaryfile1.docx]

Supplementary Material 1: Survey Items

*Inclusion Criteria*

1. Do you agree to participate in this survey? (Yes/No)
2. What is your clinical discipline?
   a. Clinical Pharmacist
   b. Dentist
   c. Kinesiotherapist
   d. Nurse Practitioner
   e. Nursing Professional (RN, LPN, etc.)
   f. Nutritionist/Dietician
   g. Occupational Therapy Practitioner
   h. Physical Therapy Practitioner
   i. Physician *[If selected: provider specialty drop-down list]*
      i. Anesthesiology
      ii. Cardiology
      iii. Critical Care
      iv. Dermatology
      v. Emergency care
      vi. Endocrinology
      vii. Gastroenterology
      viii. Geriatric medicine
      ix. Gynecology
      x. Hematology
      xi. Infectious disease
      xii. Internal medicine
      xiii. Nephrology
      xiv. Neurology
      xv. Oncology
      xvi. Orthopedics
      xvii. Otorhinolaryngology
      xviii. Palliative care
      xix. Primary care
      xx. Psychiatry
      xxi. Pulmonology
      xxii. Radiology
      xxiii. Rheumatology
      xxiv. Surgery
      xxv. Urology
      xxvi. Something else: [Open text]
   j. Physician Assistant
   k. Psychologist
   l. Recreation Therapist
   m. Social Worker
   n. Speech Language Pathologist
   o. Other Mental Health Counselor: [Open text]
   p. Other: [Open text]
   q. I am not a clinician
3. Are you a VA Fellow, Resident, or other Trainee? (Yes/No)
   a. *If yes,* “Have you been training at the VA full time (40 hours/week) for at least 1 month?” (Yes/No)
4. Do you see Veterans in an outpatient setting? (Yes/No)
5. What percentage of your care is delivered in an outpatient setting? (None; Less than 10; 10-24; 25-50; 51-99; 100)

*In-Home Video Utilization*

6. In the past 12 months, about what percentage of your care was delivered using VA Video Connect (VVC)/video-into-the-home? (None; Less than 10; 10-24; 25-50; 51-99; 100)
7. What types of clinical services do you deliver using VA Video Connect/video-into-the-home? Select all that apply, or “none.”
a. Routine assessment/examination
   i. *[If yes]* Involves physical/ cognitive evaluation
b. Assessment/examination of new or acute issue
   i. *[If yes]* Involves physical/ cognitive evaluation
c. Ongoing chronic disease management
d. Ongoing care of mental health issues (e.g., counseling or talk therapy)
e. Hospital or ED follow-up care
f. Medication management
g. Home environment assessment or observation
h. Care coordination
i. One-to-one care
j. Group treatment
k. Other [open text]
l. None
8. In general, how would you rate the quality of care you deliver over VVC/video-into-the-home compared to in the clinic? (Much Worse; Somewhat Worse; About The Same; Somewhat Better; Much Better)

*Patient Factors When Offering In-Home Video*

9. Which of the following VETERAN factors do you consider when offering VVC? Select all that apply.
a. Rurality or distance from VA
b. Travel barriers including inclement weather, lack of transportation, traffic
c. Skills with technology or technological literacy
d. Access to email, device (e.g., laptop or smartphone), or broadband internet
e. Willingness and/or preference for video
f. Functional status (ADL and IADL status)
g. Vision and hearing
h. Cognition
i. Motor and sensory function (e.g., strength, ROM, coordination, sensation)
j. Age
k. Medical acuity
l. Medical complexity
m. Psychological/psychiatric factors (e.g., mood, PTSD, substance use disorder)
n. Ability to communicate effectively
o. Availability of a caregiver to provide assistance
p. Veteran/caregiver history of successful VVC encounters
q. Other [open text]
r. None of the above

*Clinical and Administrative Factors When Offering In-Home Video*

10. How do the following CLINICAL or ADMINISTRATIVE factors influence your decision to offer VVC? (Matrix item with 4 options: Negatively/less likely to offer – Neutral/Not a Factor – Positively/more likely to offer – Not Available to Me).
a. MSA or TCT (Telehealth Clinical Technician) availability to schedule VVC
b. MSA or TCT availability to provide test calls and/or technical assistance to Veterans/caregivers
c. Number of Veterans in my panel
d. Need for frequent follow-up
e. High Veteran acuity or need to be seen in a timely way
f. Goal of care involving ability to see in home (e.g., medication management, home safety)
g. Number of available VVC/video-into-the-home appointments
h. Ability to work remotely
   i. *[If yes]* Are you a fully remote employee who only offers telehealth? (Yes/No)
i. Departmental or facility target for number of VVC encounters
j. Convenience of VVC/video-into-the-home to my workflow (e.g., ability to write notes or enter orders in real-time, use of VVC when I have an injury, or for inclement weather)
k. Workload credit
l. Environment at VA or at my home (e.g., space, equipment, broadband, privacy, and set-up)
m. Other: [Open text]

*Barriers to In-Home Video*

11. What barriers (if any) have you encountered in integrating VA Video Connect (VVC)/video-into-the-home into your practice? Select all that apply.
a. Administrative Factors
i. Lack of leadership support at supervisor or facility level for using VVC
ii. Inadequate space, physical locations, and/or related equipment
iii. Lack of administrative support (e.g., assistance with scheduling, setting up clinics, etc.)
iv. Technical challenges on the clinician/VA side
v. Other [open text]
vi. None
b. Veteran Factors
vii. Veteran/caregiver preference for coming into the clinic
viii. Veteran preference for telephone instead of video
ix. Veteran/caregiver difficulty with technology limiting their capacity to use VVC
x. Veteran/caregiver medical/functional impairments limiting their capacity to use VVC
xi. Veteran lack of access to broadband/Wi-Fi, email, or device needed for video
xii. Other [open text]
xiii. None
c. Clinician Factors
xiv. I see little added benefit to using VVC
xv. The clinical care I provide is not feasible over video
xvi. I lack the time and/or capacity to adapt my clinical procedures to video
xvii. I found the training or other set-up requirements burdensome
xviii. Other [open text]
xix. None

*Benefits to In-Home Video*

12. What are the key benefits of using VA Video Connect/video-into-the-home with Veterans? Select all that apply, or ‘none.’
    a. Enables Veterans faster access to care
    b. Eliminates the need for Veterans to travel to and from appointments
    c. Increased convenience for Veterans (e.g., reduces need to take time off work)
    d. Reduces the stress on Veterans or caregivers
    e. Reduces potentially harmful exposures (viruses, infectious diseases)
    f. Allows me to provide more comprehensive care to Veterans
    g. Enables me a view into Veterans' homes or of Veteran in-home functioning
    h. Other [Open text]
    i. None

*Frequency of Use and Effectiveness of In-Home Video Facilitators*

13. To your knowledge, how OFTEN have you or the Veterans you serve utilized the following in relation to your VVC/video-into-the home visits? (Matrix: Never; Rarely; Sometimes; Often; Always; Not available; I Don’t Know)
    a. VA National Help Desk
    b. VA tablets
    c. Reminder emails sent by me or my team member
    d. Reminder emails automatically generated by Virtual Care Manager or VVC
    e. Test call conducted by the VA National Help Desk
    f. Test call conducted by someone at my facility
    g. Test call conducted by me
    h. My assistance to the Veteran/caregiver with technical issues DURING log-in
    i. My assistance to the Veteran/caregiver with technical issues AFTER log-in
    j. Technical assistance for log-in or technical issues provided to the Veteran/caregiver by someone else at my facility
    k. Digital Divide consult
    l. VA Telehealth website (https://telehealth.va.gov/)
    m. Handouts and instructional guides provided by VA Telehealth
    n. Other: [Open text]
14. How EFFECTIVE are the following in facilitating VVC/video-into-the home visits? (Matrix: Not Effective; Somewhat Effective; Effective; Very Effective; Extremely Effective) *[Skip logic = exclude items selected “Never,” “not available”, or “unknown” in prior item] [skip logic = include for each item in #14 selected > None]*
a. VA National Help Desk
b. VA tablets
c. Reminder emails sent by me or my team member
d. Reminder emails automatically generated by Virtual Care Manager or VVC
e. Test call conducted by the VA National Help Desk
f. Test call conducted by someone at my facility
g. Test call conducted by me
h. My assistance to the Veteran/caregiver with technical issues DURING log-in
i. My assistance to the Veteran/caregiver with technical issues AFTER log-in
j. Technical assistance for log-in or technical issues provided to the Veteran/caregiver by someone else at my facility
k. Digital Divide consult
l. VA Telehealth website: https://telehealth.va.gov/
m. Handouts and instructional guides provided by VA Telehealth
n. Other (as you described above)

*Demographics*

15. How many years have you been a clinician? (Less than 1; between 1 and 5; 6-10; 11-20; 21-30; More than 30)
16. How many of those years have been at VHA? (Less than 1; between 1 and 5; 6-10; 11-20; 21-30; More than 30)
17. What is your age? (18-24 years old; 25-34 years old; 35-44 years old; 45-54 years old; 55-64 years old; 65-74 years old; 75-84 years old; 85 or older)
18. What is your sex? (Male; Female; Prefer not to answer)
