# Supplementary material for: Offering in-home video visits: a descriptive national survey of interprofessional clinicians across Veterans Health Administration
Source: Front Digit Health. 2026 Sep 14;8:1912233. doi: 10.3389/fdgth.2026.1912233 (PMC13617108; doi:10.3389/fdgth.2026.1912233)
Supplement: Supplementary file 2 [file Supplementaryfile2.docx]

Supplementary Material 2: Table. Respondents’ Clinical Disciplines and MD Sub-Specialties

|  | N | Responses, n (%) |
| --- | --- | --- |
| Clinical Discipline | 11,416 |  |
| Clinical Pharmacist |  | 496 (4.34) |
| Dentist |  | 14 (0.12) |
| Kinesiotherapist |  | 17 (0.15) |
| Nurse Practitioner |  | 1,120 (9.81) |
| Nursing Professional (RN, LPN, etc.) |  | 1,738 (15.22) |
| Nutritionist/Dietician |  | 318 (2.79) |
| Occupational Therapy Practitioner |  | 198 (1.73) |
| Physical Therapy Practitioner |  | 258 (2.26) |
| Physician |  | 2,112 (18.50) |
| Physician Assistant |  | 258 (2.26) |
| Psychologist |  | 1,220 (10.69) |
| Recreation Therapist |  | 84 (0.74) |
| Social Worker |  | 2,493 (21.84) |
| Speech Language Pathologist |  | 87 (0.76) |
| Other Mental Health Counselor |  | 271 (2.37) |
| Other |  | 732 (6.41) |
| Physician Specialty (if applicable) | 2,011 |  |
| Anesthesiology |  | 17 (0.85) |
| Cardiology |  | 40 (1.99) |
| Critical Care |  | 1 (0.05) |
| Dermatology |  | 21 (1.04) |
| Emergency Care |  | 39 (1.94) |
| Endocrinology |  | 52 (2.59) |
| Gastroenterology |  | 34 (1.69) |
| Geriatric Medicine |  | 58 (2.88) |
| Gynecology |  | 26 (1.29) |
| Hematology |  | 8 (0.40) |
| Infectious Disease |  | 57 (2.83) |
| Internal Medicine |  | 279 (13.87) |
| Nephrology |  | 44 (2.19) |
| Neurology |  | 90 (4.48) |
| Oncology |  | 31 (1.54) |
| Orthopedics |  | 28 (1.39) |
| Otorhinolaryngology |  | 12 (0.60) |
| Palliative Care |  | 39 (1.94) |
| Primary Care |  | 320 (15.91) |
| Psychiatry |  | 433 (21.53) |
| Pulmonology |  | 54 (2.69) |
| Radiology |  | 0 (0.00) |
| Rheumatology |  | 33 (1.64) |
| Surgery |  | 79 (3.93) |
| Urology |  | 24 (1.19) |
| Other |  | 192 (9.55) |
